# Direct ^14^C dating of equine products preserved in archaeological pottery vessels from Botai and Bestamak, Kazakhstan

**DOI:** 10.1007/s12520-022-01630-2

**Published:** 2022-08-18

**Authors:** Emmanuelle Casanova, Timothy D. J. Knowles, Alan K. Outram, Natalie A. Stear, Mélanie Roffet-Salque, Viktor Zaibert, Andrey Logvin, Irina Shevnina, Richard P. Evershed

**Affiliations:** 1 Organic Geochemistry Unit, School of Chemistry, University of Bristol, Cantock’s Close, Bristol, BS81TS UK; 2 Present Address: UMR7209 Archaeozoology and Archaeobotany, Centre national de la recherche scientifique/Museum National d’Histoire Naturelle, CP56 55 rue Buffon, 75005 Paris, France; 3 Bristol Radiocarbon Accelerator Mass Spectrometry Facility, University of Bristol, 43 Woodland Road, Bristol, BS81UU UK; 4 Department of Archaeology, University of Exeter, Laver Building, North Park Road, Exeter, EX4 4QE UK; 5 Institute of Archaeology and Steppe Civilizations, Al-Farabi Kazakh National University, 71 Al-Farabi St, Almaty, Kazakhstan; 6 Archaeological Laboratory, Kostanay Regional University Named After A. Baitursynov, Baitursynov St., 47, Kostanay, Kazakhstan

**Keywords:** Radiocarbon dating, Ceramics, Equine products, Kazakhstan

## Abstract

Direct and accurate radiocarbon dating of lipid residues preserved in ceramics is a recently established method that allows direct dating of specific food products and their inception in human subsistence strategies. The method targets individual fatty acids originating from animal fats such as ruminant dairy, ruminant adipose, non-ruminant adipose and aquatic fats. Horse lipid residues found in Central Asian pottery vessels are also directly dateable using this new method. Here we present the identification of equine lipid residues preserved in two pottery assemblages from the Neolithic and Eneolithic in Kazakhstan and their direct ^14^C dating. The site of Botai, previously radiocarbon-dated to the 4th millennium BC, was used as a reference to evaluate the dates obtained directly on horse lipids. The direct dating of equine products extracted from Botai potsherds are shown to be compatible with previous ^14^C dates at the site. The site of Bestamak, lacking previous^14^C measurements, had been relatively dated to the Neolithic based on pottery typologies. The direct dating of equine residues made it possible to anchor the pottery assemblage of Bestamak in the 6th millennium BC confirming their Neolithic attribution. These findings demonstrate the potential for dating horse products through a compound-specific approach, while highlighting challenges in ^14^C dating individual fatty acids from lipid extracts in which their abundances differ substantially.

**Supplementary Information:**

The online version contains supplementary material available at 10.1007/s12520-022-01630-2.

## Introduction

The exploitation of horses for their primary products was common in Central Asia during Prehistory. These were intensively processed in pottery vessels from the Botai and Tersek Eneolithic cultures, but their importance in the subsistence economy decreased from the early Bronze Age with the rise in exploitation of ruminant products (Outram et al. 2011, 2012). Horse lipid residues in ancient vessels, like other animal fats, are dominated by palmitic (C_16:0_) and stearic (C_18:0_) fatty acids (FAs) derived from the degradation of triacylglycerols (Evershed et al. 1995). However, the relative abundance of the C_16:0_ compared to the C_18:0_ FAs from fresh horse fat is higher than in any other animal species. For example, fresh modern equine product fats from Kazakhstan have an average C_16:0_/ C_18:0_ ratio for meat and milk of 7.5 ± 2.9 s.d., respectively, compared to 2.1 ± 0.9 s.d. in other animal fats (ruminants, porcine, fish), permitting discrimination of lipids of equine origin when fresh (Stear 2008; Outram et al. 2009). However, this criterion cannot be used to distinguish archaeological horse fats from other fat sources due to fatty acid distributions being altered during burial (De Jong et al. 1961; Dudd et al. 1998; Mills and White 2012; Whelton et al. 2021). Significantly, determinations of δ^13^C values of the C_16:0_ and C_18:0_ FAs offer the possibility to discriminate horse lipids from ruminant, porcine or aquatic products. Additionally, δ^2^H analyses of individual FAs enable discrimination of equine meat from equine milk (Outram et al. 2009). The latter is possible as hydrogen isotopic composition is sensitive to seasonal variations in precipitation. Mare’s milk is only exploited seasonally during the spring and summer (driest seasons of the year) with its lipids biosynthesised in the season of milk let-down, whereas meat and adipose fats undergo long-term biosynthesis and remodelling throughout the year resulting in an integrated annual signal for the animal’s FA pool. This allowed for the first-time identification of mare’s milk in pottery vessels, at Botai in the 4th millennium BC (Outram et al. 2009). Alongside evidence for horse harnessing from ‘bit wear’ damage and the presence of corral structures, this provided the earliest evidence for horse domestication (Outram et al. 2009; Gaunitz et al. 2018). Despite this compelling evidence, the question of early horse domestication at Botai is still debated (Outram et al. 2021; Taylor and Barrón-Ortiz 2021).

The direct radiocarbon dating of specific commodities permits determination of the time of their introduction in human diet and verification of their antiquity in a particular context/region while removing any taphonomic uncertainty that may exist when radiocarbon dating associated materials. Thus their direct dating is extremely important to anchor the timeframe of exploitation of specific commodities. The most common materials targeted in such an application are charred seeds, bone collagen of animals exploited for their meat, or visible food residues present on the surface of pottery (but the source(s) of their constituent C are rarely identified prior to dating (Teetaert et al. 2017; Bayliss and Marshall 2019). A new method for reliably radiocarbon dating pottery vessels from the absorbed food residues preserved within the ceramic fabric has now been validated (Casanova et al. 2020). The approach is based on the compound-specific radiocarbon analysis (CSRA) of the two most abundant FAs (C_16:0_ and C_18:0_) characteristic of degraded animal fats preserved within the vessel walls. This dating method is useful in fixing timeframes at sites where traditionally ^14^C dated materials do not survive, dating directly typologically characteristic pottery, verifying the antiquity of lipids within sherds and, of interest here, directly dating the use of specific food products (Casanova et al. 2020).

This new method has been rigorously validated using modern references, lipids from bog butter specimens and pottery assemblages from well-dated sites (Casanova et al.^ 2018^, 2020c), and then applied to various archaeological questions and sources of lipids. The CSRA method has been used on lipids of terrestrial origin, such as porcine and ruminant products, with a particular focus on the direct dating of ruminant dairy residues. Dairy residues were directly ^14^C-dated to time their inception in human food procurement, for instance, in Central Europe, the Balkans, and North Africa (Casanova et al. 2020a, c; In press; Stojanovski et al. 2020), or to verify their antiquity in a particular archaeological context (Dunne et al. 2019; Fewlass et al. 2020). Biases in ^14^C dates (reservoir effects) of lipids with a marine origin as well as their mixing with terrestrial resources have also been explored, and means of correction for the marine reservoir effects established (Casanova et al. 2020; Casanova 2022). All these studies demonstrate that the fatty acids from various food sources, recovered in pottery vessels, can be dated accurately, providing the C_16:0_ and C_18:0_ FAs survive in sufficient amounts. This suggests FAs from other sources, such as equine, are within the scope of this dating method.

Identification of horse lipid residues from food processing in pottery vessels from the Kazakh steppe (Outram et al. 2011; Outram 2012) provides a unique opportunity to apply the new CSRA method to the dating of FAs of equine origin. In this paper we focus on ceramics from the Botai settlement, for which previously measured ^14^C dates are available, to serve as a test for the dating of FAs of equine origin from a well-dated site, and the site of Bestamak (Stear 2008), lacking absolute chronology. Hence, we present here the first example of lipid residue analyses of pottery with the identification of horse products followed by their direct ^14^C dating.

## Materials and methods

### Botai

Botai (67.645786 E, 53.303942 N) is an Eneolithic site of the 4th millennium BC in Northern Kazakhstan, which gave its name to a culture recognised for intensive horse exploitation and early forms of domestication. The settlement comprises ca. 300 pit house dwellings (Levine and Kislenko 1999). Faunal assemblages are abundant (300,000 bones), dominated by horse remains (99.9%) with some wild animal remains including aurochs, bison, antelope and marmot. This site presents several lines of evidence for the husbandry of horses (Olsen 2006; Outram et al. 2011; Outram and Bogaard 2019). For this study, pottery vessels of Eneolithic typologies at the Botai settlement (*n*= 89) previously analysed for their lipid residues (Outram et al. 2011) were considered for ^14^C dating.

For Botai, a total of 25 radiocarbon dates on horse bone collagen, human collagen and botanical remains are available in the literature, which estimates the Eneolithic phase to ca. 3600–3100 BC (Levine and Kislenko 1997; Levine 1999; Outram et al. 2009; De Barros Damgaard et al. 2018; Gaunitz et al. 2018; Mortuzaite Matuzeviciute et al. 2019). The earliest published dates, however, exhibit rather large uncertainties (Levine and Kislenko 1999; Levine 1997) compared to more recent ones. Stratigraphical sequences have not been highlighted, thus, the relative relationship between dated materials is not well-established. Only the ^14^C dates on botanical remains from a particular house have been modelled using Bayesian statistics (Motuzaite Matuzeviciute et al. 2019).

### Bestamak

Bestamak (64.724468 E, 51.575646 N) is a multi-period complex of sites in the Turgai Plateau comprising a settlement and cemetery. It has phases dating from the Neolithic through to the Iron Age. The Bronze Age cemetery has been ^14^C-dated and studied in relation to the diet of the human population (Ventresca Miller et al. 2014; Ventresca 2018). Horse bones from the Eneolithic, Middle Bronze Age and Iron Age phases have been ^14^C dated as part of an ancient DNA study (Librado et al. 2021). The phase with typologically dated Neolithic material culture, however, lacks absolute dating. This multiple phase site has yielded ceramics of the Neolithic, Eneolithic (Tersek culture) and Bronze Age phases. For this study, pottery vessels of the Makhandzhar Neolithic culture at the Bestamak settlement (*n* = 38) were selected for lipid residue analyses and ^14^C dating.

### Protocol for lipid residue analyses

Lipid residue analyses in pottery were performed following established procedures (Evershed et al. 1990; Charters et al. 1993). In brief, ~ 2 g of each potsherd surface was cleaned then ground to a fine powder before being transferred to a glass vial along with 20 μL of internal standard (*n*-tetratriacontane). Lipids were extracted by sonication in a chloroform/methanol solution (10 mL; 2:1 *v/v*, 2 × 20 min), then centrifuged (2500 rpm, 10 min). The supernatant comprising the total lipid extract (TLE) was filtered through a silica column (1 g) to remove any particulate matter.

An aliquot of the TLE was treated with *N,O*-bis(trimethylsilyl)trifluoroacetamide (BSTFA) containing 1% trimethylchlorosilane (40 μL; 70 °C, 1 h) with excess removed under N_2_ stream, and was dissolved in *n*-hexane prior to high temperature gas chromatography (HTGC) and HTGC/mass spectrometry (MS) analyses (Supplementary information (SI) 1; Stear 2008).

A second aliquot of the TLE was hydrolysed using a methanolic sodium hydroxide solution (5% *v/v*, 70 °C, 1 h) with regular mixing. The neutral fraction was extracted with *n*-hexane (3 × 3 mL). The remaining solution was acidified to pH 3 (HCl 1 M) and the free fatty acids were extracted with chloroform (3 × 3 mL) and excess solvent removed under nitrogen. Fatty acid methyl esters (FAMEs) derivatives of FAs were prepared with BF_3_-methanol (14% *w/v*, 100 μL, 70 °C, 1 h). Double distilled water was added (1 mL), the FAMEs extracted with chloroform (3 × 2 mL) and the solvent removed under nitrogen. FAMEs were re-dissolved in *n-*hexane before analysis by GC and GC-combustion-isotope ratio mass spectrometer (GC-C-IRMS; SI 1; Stear 2008). GC-C-IRMS analyses were performed to measure δ^13^C values on individual C_16:0_ and C_18:0 _FAs (SI 1; Stear 2008; Outram et al. 2011).

### Protocol for radiocarbon dating and calibration

The CSRA method was performed using an acidified solution of methanol to extract lipids preserved in pottery (Casanova et al. 2020c), a method about five times more efficient than the solvent extraction used to pre-screen for lipid preservation in this study (Correa-Ascencio and Evershed 2014). For ^14^C dating, potsherds with lipid concentrations over 100 µg of lipid per g of sherd were selected (usually > 500 µg·g^−1^ using the acidified methanol solution) and *c*. 5 g were sampled to maximise the chances of obtaining enough carbon (*c*. 200 µg of C per fatty acid). The radiocarbon determinations were thus performed on separate TLEs than those used for lipid residue analysis.

Lipids were extracted and methylated using an acidified methanol solution (H_2_SO_4_/MeOH, 4% *v/v*, 3 × 8 mL, 1 h, 70 °C). The extracts were centrifuged (10 min, 2500 rpm), and the supernatants combined in a culture tube. Lipids were extracted by liquid–liquid extraction with *n*-hexane (4 × 5 mL) then transferred to small vials and blown down to dryness. A procedural blank was performed with every batch to monitor (by GC analysis) potential contamination arising from the lipid extraction step. The FAMEs (re-dissolved in sufficient hexane to achieve a concentration of ~ 5 μg·μL^−1^ for the C_18:0_ FA) were isolated 40 times in individual solventless traps using a preparative capillary gas chromatography (pcGC) instrument (Casanova et al. 2020b, c). This step allows removal of exogenous contaminants from the burial environment or the chemical pre-treatment (excluding contaminants that would co-elute with the FAMEs). Pseudo-processing standards, i.e. *n-*hexane injections to mimic the trapping of the FAMEs, then addition of IAEA-C7 oxalic acid standard or phthalic anhydride blank (Casanova et al. 2020b, c), were also prepared alongside each batch of samples to monitor for potential contamination from the pcGC isolation.

Isolated lipids (on glass wool) were transferred to an Al capsule for combustion in an elemental analyser (VarioCube) linked to an automatic graphitisation equipment (AGE3, Ionplus) prior to being measured on the BRIS-MICADAS instrument (Knowles et al. 2019). The graphitised lipids were measured in several batches based on their C mass. Size-matched standards (Oxalic Acid II, IAEA-C7) and blanks (phthalic anhydride), including the pseudo-processing standards, were analysed alongside each batch of FAMEs isolated from pottery vessels for normalisation and background corrections (Casanova et al. 2020b; Knowles et al. 2019).

The ^14^C measurements on FAMEs were corrected for the derivative carbon using a simple mass balance approach as fractionation during the methylation of FAs has been shown to be negligible (Stott et al. 2003; Casanova et al. 2020b); Casanova 2019). As described in Casanova et al. (2020c), the two FAs dated (C_16:0_ and C_18:0_) are from the same source, and their statistical equivalence (at the 5% level) provides an internal quality control measure for the potsherds dated. If this quality control test was passed, the C_16:0_ and C_18:0_ FA ^14^C measurements were combined as described in Casanova et al. (2020c) before calibration.

The radiocarbon dates were calibrated against the northern hemisphere atmospheric calibration curve Intcal20 (Reimer et al. 2020) in OxCal v4.4 (Bronk Ramsey 2009). Due to the lack of stratigraphy at the site, existing ^14^C dates from Botai were modelled in a single phase, and charcoal samples treated as *termini post quem* (TPQ) dates to mitigate for potential old wood effects.

## Results and discussion

In this section, we present the ^14^C measurements obtained from the site of Botai and Bestamak (Table 1) and how they fit the local chronologies, the required methodological considerations for CSRA of equine lipid dating, and the significance of the dates for Eurasian Steppe archaeological studies.

**Table 1:** Lipid characterisation of equine products from Botai and Bestamak and conventional radiocarbon dates with lipid concentration (C°), stable carbon isotopes on individual fatty acids (δ^13^C_16:0_, δ^13^C_18:0_), CSRA dates on individual fatty acids, their combined measurement and *T* test values

Pot #	C° (µg/g)	Ratio C_16:0_/C_18:0_	δ^13^C_16:0_ (‰)	δ^13^C_18:0_ (‰)	BRAMS #	C_16:0_ age (BP)	C_18:0_ age (BP)	Combined age (BP)	T’
BOT7	254	5.3	− 27.8	− 27.7	BRAMS-3043	4460 ± 27	4738 ± 29	-	33.2
BOT26	606	13.3	− 27.3	− 27.7	BRAMS-3044.1 BRAMS-3044.2	4839 ± 27 4478 ± 25	6730 ± 26 4399 ± 33	- 4465 ± 22	1499 2.4
BOT3	583	4.4	− 27.7	− 28.3	BRAMS-3045	4459 ± 27	4525 ± 29	4485 ± 23	1.9
BOT18	510	3.7	− 26.7	− 27.0	BRAMS-3047	4507 ± 27	4415 ± 29	4471 ± 23	3.8
BOT24	554	5.0	− 28.0	− 27.9	BRAMS-3046	4669 ± 27	4679 ± 27	4674 ± 22	0.1
BOT10	440	5.3	− 27.9	− 27.9	BRAMS-3048	4511 ± 27	4609 ± 29	-	4.0
BOT70	554	3.9	− 29.3	− 29.1	BRAMS-3049.1	4636 ± 27	4839 ± 31	-	14.9
					BRAMS-3049.2	4630 ± 33	-	-	-
PS460	95	6.6	− 28.4	− 29.3	BRAMS-3050	6216 ± 27	-	-	-
PS466	166	4.6	− 28.3	− 37.7	BRAMS-3051	6272 ± 27	-	-	-
PS484	247	1.5	− 23.0	− 22.7	BRAMS-3052	6052 ± 27	6115 ± 25	6092 ± 22	1.6

### Botai

**Fig. 1:**
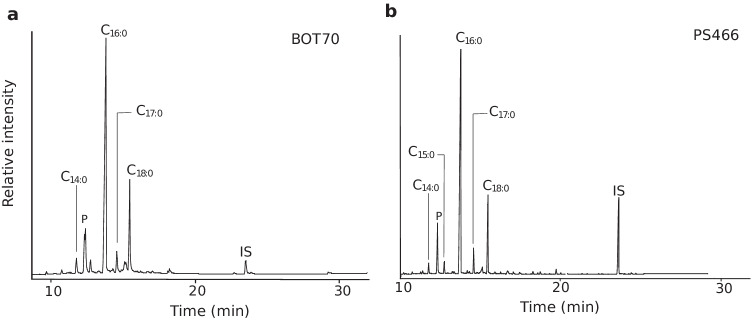
Partial gas chromatogram of a TLE from **a** Botai and **b** Bestamak showing lipid distributions typical of animal fats. IS is the internal standard (*n*-tetratriacontane), P is plasticiser (modern contamination), C_n:0_ are saturated fatty acids with n carbon atoms

Lipid residues analyses from this pottery assemblage have been reported in an earlier publication (Outram et al. 2011) and are briefly described here (SI TableS1). A total of 82% (*n* = 73) of the TLEs showed lipids were present in appreciable concentrations (> 5 µg·g^−1^) with an average lipid concentration of 391 µg·g^−1^and maximum of 3.7 mg·g^−1^. All the TLEs are dominated by C_16:0_ and C_18:0_ FAs. The C_16:0_ and C_18:0_ ratios for 27% of the TLEs (*n* = 18) are above 4, strongly suggesting an equine origin but as discussed before this criterion by itself is not a reliable species indicator (Fig. 1a). A total of 14% (or *n* = 12) of the sherds contained the C_31_, C_33_, C_35_ long-chain ketones characteristic of the heating of vessels content to high temperature (Evershed et al. 1995). Most δ^13^C_16:0_ and δ^13^C_18:0_ values are characteristic of equine fats products (*n* = 45 or 84%) which is consistent with the dominance of horse remains in the faunal assemblage (Fig. 1b). A total of *n* = 5 sherds display δ^13^C values characteristic of ruminant adipose fats which could originate from wild aurochs or antelopes, although we should note that two of these residues have δ^13^C values that are close that those expected for equine fats, and thus could also be of equine origin.

**Fig. 2:**
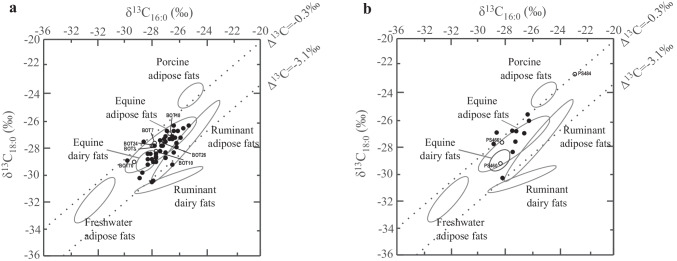
δ^13^C_18:0_ values plotted against the δ^13^C_16:0_ values for animal fats from potsherds from the sites of **a** Botai and **b**Bestamak. Ellipses correspond to modern references values from Kazakhstan (Outram et al. 2011). Horse adipose and dairy fats ellipses completely overlap meaning these two products cannot be discriminated using δ^13^C_16:0_ and δ^13^C_18:0_ values. Animal fats selected for ^14^C dating as part of this study are displayed using open circles and labelled with their sample names

For radiocarbon dating, we selected potsherds with residues clearly identified as equine products (*n* = 7) based on their δ^13^C values. It should be noted the relative abundance of the C_16:0_ FA for these extracts is between 3 and 5 times higher than the C_18:0_ FA. Thus, the mass of C in the cathodes after the graphitisation differed greatly for the two FAs extracted from the same potsherds. These required measurement in separate analytical sequences on the AMS, with sized-matched standards for the specific targets. The concentrations of the samples isolated on the pcGC were adjusted to ensure that ~ 200 µg of the least abundant FA was isolated. In some cases, this would result in over 1 mg of the C_16:0_ FA being isolated. Because sample sizes are limited to 1 mg during graphitisation, this would affect the apparent blank contribution from the pcGC isolation and sample combustion procedures. To avoid this, the C_16:0_ FA from some lipid extracts (based on their relative concentrations) was only collected from only ten pcGC runs, while the C_18:0_ FA was isolated from 40 runs. After the first ten injections, the trap containing the isolated C_16:0_ FA was removed and replaced with a new one and the remaining 30 injections required to isolate a sufficient amount of the C_18:0_ FA were performed. This is the first time we have used such an approach due to the difference in the relative abundances of the C_16:0_ and C_18:0_ FAs. In previous dating programs involving ruminant fats, lipid extracts with similar abundances of the two FAs were considered the best candidates for dating (e.g. Casanova et al. 2018; 2020a, c; Casanova 2022; In press) (Fig.2).

Four of the equine adipose fats exhibited statistically consistent ages between the C_16:0_ and C_18:0_ FAs radiocarbon dates (Table 1) which were combined as described in Casanova et al. (2020c) to give 4465 ± 22 BP (BOT26, BRAMS-3044), 4485 ± 23 BP (BOT3, BRAMS-3045), 4674 ± 22 BP (BOT24, BRAMS-3046) and 4471 ± 23 BP (BOT18, BRAMS-3047). Lipids in potsherd BOT26 were dated twice as the first^14^C measurements clearly showed contamination of the C_16:0_ target by fossil carbon (likely from graphite ferrule degradation in the pcGC), which exhibited older ages by 2000 ^14^C years. This was resolved in the repeated analysis, and only the latter measurement was used in this study. The TLEs which exhibited the highest C_16:0_/C_18:0_ FA ratios unfortunately failed the internal quality control test on the statistical consistency of the C_16:0_ and C_18:0_ FA ^14^C measurements. A second isolation was only possible for BOT70 but we could only obtain a date on the C_16:0_ FA due to the lower abundance of C_18:0_ FA. Furthermore, the C_18:0_ FA only yielded 100 µg of C on the first isolation and this is below the tested limit of reliability (200 µg) for precise and accurate ^14^C measurements on single compound using graphite targets (Casanova et al. 2020c). The two C_16:0_ FA ages of potsherd BOT70 are statistically consistent (T’ = 0.0, T’ (0.05) = 3.8, ν = 1) and give a combined age of 4634 ± 21 BP. This supports the conclusion that the age determined for the C_18:0_ FA from the first extraction was erroneous and likely contaminated during the isolation procedure.

**Fig. 3:**
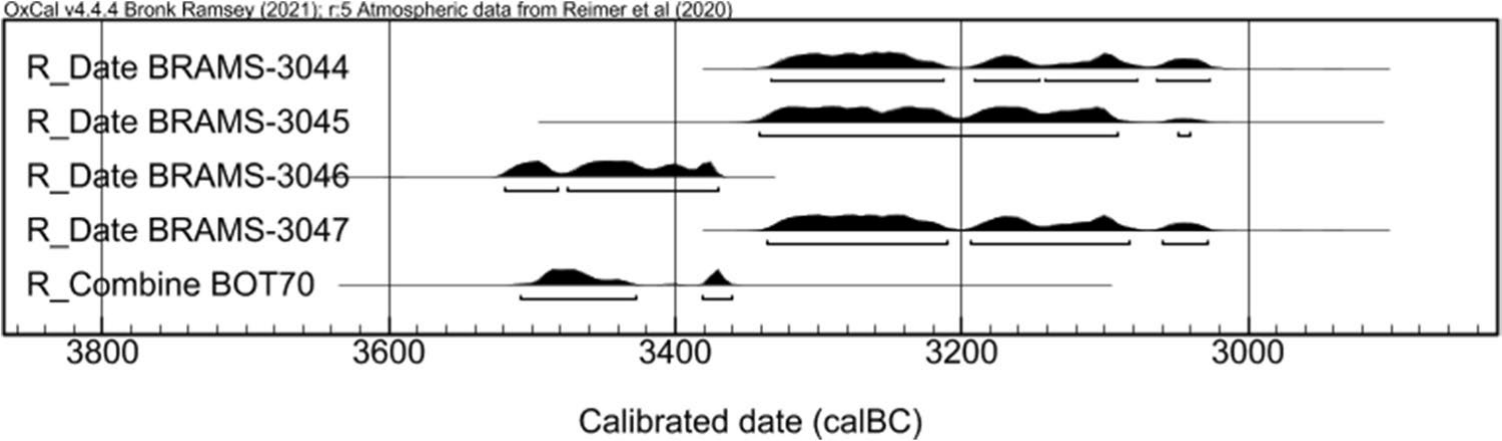
Calibrated ranges for the Botai ^14^C dates on horse lipids

The dated potsherds fell into two clear age ranges (Fig. 3). BRAMS-3046 (BOT24) and combined measurements for BOT70 are statistically consistent (T’ = 1.7, T’ (0.05) = 3.8, ν = 1) and calibrate between the mid-36th and mid-34th century BC (Fig. 3). BRAMS-3044 (BOT26), BRAMS-3045 (BOT3) and BRAMS-3047 (BOT18) are statistically identical (T’ = 0.4, T’ (0.05) = 9.1, ν = 2) and calibrate between the mid-34th and late 31st century BC (Fig. 3).

We compiled the 25 radiocarbon dates on horse bone collagen, human collagen and botanical remains already published (SI Table S2) to create a single-phase model in OxCal as reference (Levine and Kislenko (Levine and Kislenko 1997; Levine 1999; Outram et al. 2009; de Barros Damgaard et al. 2018; Gaunitz et al. 2018; Motuzaite Matuzeviciute et al. 2019). Charcoal dates were treated as TPQs dates, which is common practice to mitigate for any samples with a potential old wood effect. Two of the dates (IGAN-4234 and IGAN-449) had poor agreements in the sequence and were treated as outliers. The revised model has a good agreement ([A = 108]) and suggests the Botai sequence started in*3660–3423* cal BC (95% probability) probably in *3568–3469* cal BC (68% probability) and ended in *2887–2673* cal BC (95% probability) probably in *2873–2783* cal BC (68% probability; SI Figure S1).

**Fig. 4:**
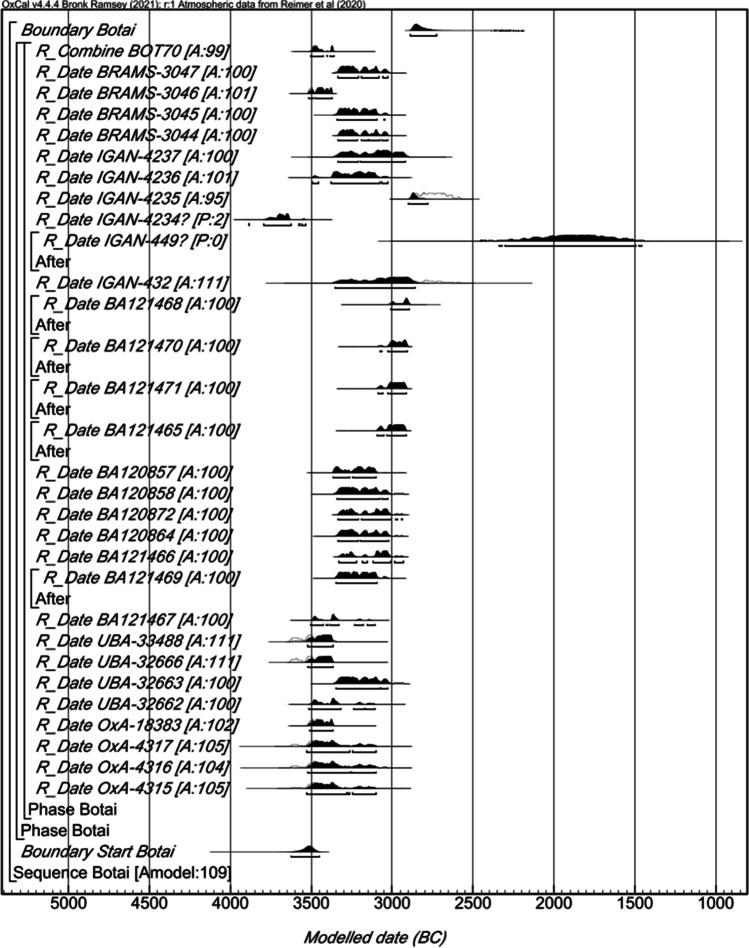
Probability distribution of CSRA dates from lipid residues diagnostic of horse products in pottery (BRAMS laboratory code) and published ^14^C dates for Botai. Each distribution represents the relative probability that an event occurs at a particular time. For each of the dates, two distributions have been plotted: one in outline, which is the result of simple radiocarbon calibration, and a solid one, based on the chronological model used. Distributions other than those relating to particular samples correspond to aspects of the model. The distribution followed by ‘?’ has been excluded from the model. The large square brackets down the left-hand side of the figure, along with the OxCal keywords, define the overall model exactly

The dates on pottery were then added to this model. All the dates demonstrated a good agreement ([A = 109]; Fig. 4). The new model including the additional pottery FA dates suggests the Botai sequence started in *3627–3451* cal BC (95% probability) probably in *3550–3480* cal BC (68% probability) and ended in *2889–2723* cal BC (95% probability) probably in *2875–2806* cal BC (68% probability; Fig. 3). The pottery dates on horse products fit well with the pre-existing dates, and do not significantly change the estimated intervals for the start and end of the Botai sequence, supporting their compatibility and accuracy.

### Bestamak

Pottery from the Neolithic typologies at Bestamak (*n* = 38) was analysed for their lipid residues (SI Table S3). A total of 63% of the TLEs from Bestamak pottery showed lipids in appreciable concentrations (> 5 µg·g^−1^) with an average lipid concentration of 52.7 µg·g^−1^. All of the TLEs presenting residues (except six containing appreciable plasticiser contamination) are dominated by the C_16:0_ and C_18:0_ FAs (Fig. 1). The relative ratios of C_16:0_/C_18:0_ FAs are between 2 and 8, suggesting the processing of equine products in some potsherds. Most of δ^13^C_16:0_ and δ^13^C_18:0_ values measured (*n*= 11/13 or 85%) are consistent with those from modern equine products from Kazakhstan (Outram et al. 2011). One TLE (PS489) exhibited δ^13^C values consistent with either equine fats or ruminant adipose fats. Another TLE (PS484) displayed relatively enriched δ^13^C_16:0_ and δ^13^C_18:0_ values compared to any of the reference values of animal fats recorded from the region; however, its Δ^13^C (= δ^13^C_18:0_ − δ^13^C_16:0_) value of 0.3‰ suggests an equine origin.

Only three sherds were considered to be datable from their lipid concentrations (> 100 µg·g^−1^) and sherd sizes (at least 5 g available for sampling). One sherd was dated to 6092 ± 22 BP (PS484, BRAMS-3052; Table 1). The other two only provided ages on the C_16:0_ FA due to the low abundance of the C_18:0_ FA in the TLEs, preventing the collection of enough C for ^14^C dating; therefore, the internal quality control criterion is not available for these dates (Table 1). The C_16:0_ FA in the residues date to 6216 ± 27 BP (PS460, BRAMS-3050) and 6272 ± 27 BP (PS466, BRAMS-3051). Again, these ^14^C dates highlight the challenges faced when working with highly different abundances of the two fatty acids. These two dates are statistically identical (T’ = 2.2, T’ (0.05) = 3.8, ν = 1) providing confidence in their accurate dating. They are nonetheless significantly older than BRAMS-3052 suggesting a rather longer period of occupation at Bestamak.

**Fig. 5:**
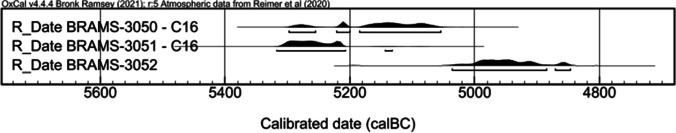
Calibrated ranges for the Bestamak ^14^C dates of horse lipids

The calibrated dates on horse products range between the 54th and 49th century BC (Fig. 4). The radiocarbon dates obtained here are consistent with the relative dating of pottery based on their typologies. Three radiocarbon dates on collagen and ceramics are available for the Makhandzhar ceramic phase, but are from other sites than Bestamak (Shevnina and Logvin 2020). The pre-existing dates (KI-13751 = 5910 ± 70 BP, SPb-1670 = 5662 ± 120 BP and SPb-1671 = 5966 ± 120 BP) present a wider uncertainty and display younger calibrated ages than the dates obtained in this study (Shevnina and Logvin 2020). These new dates on lipids preserved in Makhandzhar ceramics suggest this culture was already established in the late 6th millennium BC, and had older ancestry than the ^14^C dates from other settlements with this pottery typology suggest. Direct dating of lipid residues within potsherds provided a unique opportunity to date the Neolithic layer with Makhandzhar ceramics of the site of Bestamak. The ^14^C dates suggest a settlement persisted over several centuries at Bestamak. A more extensive dating program would be required to further refine this site chronology (Fig. 5).

## Methodological considerations

From a technical point of view, direct compound-specific ^14^C dating of archaeological horse fat residues highlighted a number of challenges in the application of the approach. The differing abundances of C_16:0_ and C_18:0_ FAs in equine residues inevitably yielded significantly different amounts of C for each of the two FAs. This was particularly problematic for the potsherds with the largest difference in relative abundances of the two FAs, especially for sherds with relatively low lipid concentrations (< 100 µg of lipids per gram of sherd). Generally, it was not problematic to isolate the most abundant C_16:0_ FA in sufficient quantities > 1 mg of C to generate a full size target for AMS measurement; however, difficulties were commonly encountered in obtaining 200 µg of C from the C_18:0_ FA required for accurate and precise CSRA dating (Casanova et al. 2020c). In order to avoid this, especially to ensure no more than 1 mg of C for each FA was collected, the number of trapping runs was reduced to 10 for the C_16:0_ FA while 40 runs were performed for the C_18:0_ FA. We were also careful to use size-matched standards and appropriate blank corrections for the two FAs extracted from the same potsherd extract. Although not all potsherds could be successfully dated using this approach, for Botai, very good agreement was obtained for the ^14^C ages for the C_16:0_ and C_18:0_ FAs, which were entirely compatible with previously published dates for the settlement, supporting the accuracy achievable using the approach. The internal control achievable by comparing the dates for the C_16:0_ and C_18:0_ FAs shows again its usefulness for verifying the accuracy of dates using this approach. The ^14^C dating success rate for this investigation was 62% (*n*= 5 out of 8, excluding potsherds dated by one target only), which contrasts with the 80–100% success rate achieved in previous studies (Casanova et al. 2020c; Stojanovski et al. 2020). This highlights that tackling samples with uneven abundance of C_16:0_ and C_18:0_ fatty acids in the extracts remains challenging. Since completing the work described here, we have successfully dated additional potsherds displaying similarly unequal abundances of FAs in pottery from the Altai (Makarewicz et al., in prep) and Niger (Manning et al., in prep) using appropriate size-matched standards and blank corrections. These studies add further confidence in the modified approach we used for CSRA in cases of uneven abundances of C_16:0_ and C_18:0_ FAs from the same potsherds.

## Significance of the radiocarbon dates on equine products in the Eurasian Steppe context

Returning to the focus of the work described here for the Eurasian Steppe, particular attention has been given to reconstructing dietary practices at Eneolithic and Bronze Age settlements by analysing food residues in potsherds (Outram et al. 2011; Outram 2012) but much less attention has been given to the Neolithic peoples who first used ceramics in the region. Therefore, exploitation of equine products in central Asia are less well documented for the earlier periods.

The new radiocarbon dates on Botai sherds proved entirely compatible with the pre-existing dates from the 4th millennium BC. Based on the dominance of horse remains at this site, and the wide processing of equine products in pottery vessels, horses have been shown to be an essential part of the economy. Direct CSRA dating of equine products from pottery vessels at Botai confirmed their Eneolithic antiquity. This is particularly significant as Botai is the only site where evidence of mare’s milk processing in pottery vessels has been reported (Outram et al. 2011). The new CSRA dates provide therefore further confidence in the ancestry of equine adipose and milk products exploitation in the 4th millennium BC. The site of Botai remains the site with the earliest domestic horse lineage evidence; however, it is not the ancestor of modern domestic breeds (DOM2) whose lineage can be traced back into the 3rd millennium BC and spread widely in the 2nd millennium BC replacing other lineages (Librado et al. 2021).

The new dates for Bestamak potsherds confirm the Neolithic origin of the Makhandzhar ceramics from the 6th millennium BC. This constitutes some of the oldest pottery yet analysed for their lipid residues in Central Asia. Animal bone assemblages associated with the Neolithic phases at Bestamak are very limited making the dating and dietary information that can be obtained from lipid residue analyses particularly useful. The lipids preserved in pottery vessels clearly indicate significant consumption of horse products at the site from the 6th millennium BC. A question remains as to whether these products would have come from wild horse hunting or even earlier husbanded horses. A way to answer this would be to determine δ^2^H values on the FAs identified as equine product and identify if some of them have δ^2^H values characteristic of mare’s milk fats.

Future analyses of a larger set of well-contextualised Neolithic sherds with δ^13^C and δ^2^H determinations would be required to demonstrate if equine milking, thus equine domestication, had earlier origins than Botai. Such a programme of research would also help reconstruct the sequence of subsistence practices that led from mixed hunting and gathering in the earlier Holocene to the extremely horse-specialised economies of the Eneolithic period in Kazakhstan.

## Conclusions

This is the first time CSRA measurements have been performed on archaeological FAs of equine origin. Working with very different abundances of FAs from the same potsherds required some methodological adjustments for appropriate corrections of ^14^C measurements. Our study nonetheless showed that equine residues are, like lipid residues from ruminant products, datable by CSRA of individual FAs. The new CSRA dates particularly resolved the absolute chronology of Bestamak ceramics which were previously only relatively constrained based on ceramic typology. Our new dates on individual FAs of equine origin from ceramics from the Eurasian steppe are entirely compatible with the Botai chronology in the 4th millennium BC, and the Neolithic period at Bestamak in the 6th millennium BC.

## Supplementary Information

Below is the link to the electronic supplementary material.

Supplementary file1 (DOCX 142 KB)
