# Supplementary material for: Direct 14C dating of equine products preserved in archaeological pottery vessels from Botai and Bestamak, Kazakhstan
Source: Archaeol Anthropol Sci. 2022 Aug 18;14(9):175. doi: 10.1007/s12520-022-01630-2 (PMC9388454; doi:10.1007/s12520-022-01630-2)
Supplement: Supplementary file 1 — Supplementary file1 (DOCX 142 KB) [file 12520_2022_1630_MOESM1_ESM.docx]

**Supplementary information**

**Direct ^14^C dating of equine products preserved in archaeological pottery vessels from Botai and Bestamak, Kazakhstan**

Emmanuelle Casanova^1,*^(ORCID 0000-0003-1417-3060), Timothy D.J. Knowles^1,2^ (ORCID 0000-0003-4871-5542), Alan K. Outram^3^ (ORCID 0000-0003-3360-089X), Natalie A. Stear^1^, Mélanie Roffet-Salque^1^ (ORCID 0000-0001-9508-788X), Viktor Zaibert^4^, Andrey Logvin^5^, Irina Shevnina^5^, and Richard P. Evershed^1,2^ (ORCID 0000-0002-9483-2750)

**^1^**Organic Geochemistry Unit, School of Chemistry, University of Bristol, Cantock’s Close, Bristol, BS8 1TS, UK,

**^2^**Bristol Radiocarbon Accelerator Mass Spectrometry Facility, University of Bristol, 43 Woodland Road, Bristol, BS8 1UU, UK

**^3^**Department of Archaeology, University of Exeter, Laver Building, North Park Road, Exeter, EX4 4QE, UK

^4^ Institute of Archaeology and Steppe Civilizations, Al-Farabi Kazakh National University, 71 Al-Farabi St., Almaty, Kazakhstan

^5^ Laboratory for Archaeological Research, Faculty of History and Law, Kostanay State University, Kostanay, Kazakhstan.

*present address: UMR7209 Archaeozoology and Archaeobotany, Museum National d’Histoire Naturelle, CP56 55 rue Buffon, 75005, Paris, France

**SI 1: Instruments and data processing for lipid residue analyses (from Stear 2008)**

**1. High temperature gas chromatography (HTGC)**

All HTGC analyses were performed on a Hewlett Packard 5890 series II gas chromatograph coupled to an Opus V PC with HP Chemstation software. One microlitre of the derivatised and diluted extract was injected onto a fused silica capillary column coated with a dimethyl polysiloxane stationary phase (DB1-HT, 15 m x 0.32 mm i.d., 0.1 μm film thickness, J&W Scientific). The temperature program consisted of 2 min isothermal at 50^o^C followed by an increase to 350^o^C at a rate of 10^o^C min^-1^. Following this, the temperature was held at 350^o^C for 10 min. Hydrogen was the carrier gas with a column head pressure of 10 psi.

**2. Gas chromatography**

FAMEs (fatty acids methyl esters) were analysed by GC using a Hewlett Packard 5890 series II gas chromatograph coupled to a PC with Clarity software. One microlitre of the derivatised and diluted extract was injected onto a fused silica capillary column coated with a high cyanopropyl modified methyl polysiloxane stationary phase (VF23ms, 60 m x 0.32 mm i.d, 0.15 μm film thickness; Varian Inc., USA). The temperature program consisted of 1 min isothermal at 50°C followed by an increase to 100°C at a rate of 15°C min^-1^ followed by an increase to 240°C at a rate of 4°C min^-1^ followed by a final increase to 260°C at a rate of 15°C min^-1^ with an isothermal period of 15 min at 260°C. Hydrogen was the carrier gas with a column head pressure of 10 psi.

**3. Gas chromatography-mass spectrometry (GC-MS)**

FAMEs were analysed by GC/MS using a ThermoFinnigan Trace MS. Samples were introduced using a PTV injector set to splitless mode onto a polydimethylsiloxane column (ZB-1, 60 m x 0.32 mm i.d, 0.1 μm film thickness, Phenomenex). The GC program consisted of an isothermal period of 2 min at 50^o^C followed by an increase to 300^o^C at a rate of 10^o^C min^-1^. Following this the temperature was held at 300^o^C for 10 min. The MS was operated in electron ionisation (EI) mode with a GC interface temperature of 300^o^C and a source temperature of 200^o^C. The emission current was 150 μA and the MS acquired in the range of *m/z* 50-650 at 1.3 scans per second. The data acquisition and processing were carried out using XCalibur software and NIST mass spectral library.

**4. High temperature GC-MS (HTGC-MS)**

Trimethylsilylated TLEs were analysed using a Perkin Elmer Turbomass Gold equipped with a fused silica capillary column (DB1-HT; 15 m x 0.25 mm i.d.; 0.1 μm film thickness; J&W). Helium was the carrier gas and the GC oven was programmed as follows: 2 min isothermal at 50^o^C followed by an increase to 350^o^C at a rate of 10^o^C min^-1^, following this, the temperature was held at 350^o^C for 10 min. The mass spectrometer was operated in full scan mode (50 – 850 daltons, 1 scan 0.6 s^-1^; 70 eV electron energy and ionisation source temperature of 240^o^C). The data acquisition and processing were carried out using XCalibur software and NIST mass spectral library.

**5. GC-combustion-isotope ratio MS (GC-C-IRMS)**

Carbon GC-C-IRMS analyses were performed using a Varian 3400 GC coupled to a Finnigan MAT Delta S IRMS via an extensively modified Finnigan MAT type I combustion interface, Cu and Pt wires (0.1 mm o.d) in an alumina reactor (0.5 mm i.d). The reactor temperature was maintained at 860°C and the mass spectrometer source pressure was 6 x 10^-6^ mbar. The GC column was a Factor Four VF-23ms column (Varian Chrompack 60 m x 0.32 mm i.d, 0.15 μm film thickness).

Analyses were also performed using a ThermoElectron Deltaplus XP isotope ratio mass spectrometer coupled to a ThermoElectron Trace GC with a Thermo-Electron GCCIII combustion interface. Samples were injected via a PTV injector in splitless mode. The GC was fitted with a fused silica capillary column (60mx0.32mm i.d) coated with a high cyanopropyl modified methyl polysiloxane stationary phase (Varian Inc., USA; VF23ms; 0.15μm film thickness). The combustion reactor temperature was maintained at a temperature of 940°C and the reduction reactor at a temperature of 600°C.

The temperature program for both instruments consisted of an isothermal period of 1 min at 40°C followed by an increase to 240°C at a rate of 10°C min^-1^ followed by an isothermal period of 10 min at 240°C. Faraday cups were used for the detection of ions of *m/z* 44, 45 and 46.

The ^13^C/^12^C ratios are expressed relative to the VPDB (*Belemnitella americana*) standard.

$\delta^{13}C=\frac{R_{sample}-R_{standard}}{R_{standard}}\times1000$ (Equation 1)

Where:

δ^13^C is measured in ‰ and

R_sample_ = ^13^C/^12^C in the sample

R_standard_ = ^13^C/^12^C in the standard

Samples were run in duplicate and any questionable runs were repeated. An external standard consisting of a mixture of FAMEs (C_11:0_, C_13:0_, C_16:0_, C_21:0_ and C_23:0_) of known isotopic composition, was run regularly between sample runs to ensure the integrity of the data. Results were calibrated against a reference CO_2_ standard, which was injected directly into the ion source three times at the beginning and three times at the end of each run. Instrumental precision was typically ± 0.3 ‰ or better. δ^13^C values for the individual fatty acids were determined by correcting the values obtained for the corresponding FAMEs using a simple mass balance calculation to account for the extra carbon added during derivatisation (Eqn. 2., Rieley, 1994)

$\delta^{13}C_{FA}=\frac{({no.C}_{FAME}\times\delta^{13}C_{FAME}{)-\delta}^{13}C_{MeOH}}{{no.C}_{FA}}$ (Equation 2)

Where:

δ^13^C_FA_= δ^13^C value of the fatty acid

δ^13^C_FAME_ = δ^13^C value of the FAME

δ^13^C_MeOH_ = δ^13^C value of the derivatising methanol

no.C_FAME_ = total number of carbon atoms in the FAME

no.C_FA_ = total number of carbon atoms in the original fatty acid

Table S1: Result table from molecular and isotopic analyses of pottery residues from Botai (Stear 2008).

| **Sample ID** | **Site ID** | **Concentration (µg.g^-1^)** | **C_16:0_/C_18:0_ ratio** | **TAGs** | **Ketones** | **δ^13^C_16:0_ (‰)** | **δ^13^C_18:0_ (‰)** | **Δ^13^C (‰)** | **δ^2^H_16:0_ (‰)** | **δ^2^H_18:0_ (‰)** | **Assignment** |
| --- | --- | --- | --- | --- | --- | --- | --- | --- | --- | --- | --- |
| BOT1 | 370ϭ-II/5694 | 136.5 | 2.78 | - | - | -26.2 | -27.7 | -1.1 | -271 | -212 | Equine adipose |
| BOT2 | 370ϭ-IV/4155+4154 | 28.5 | - | - | - | - | - | - | - | - | Contamination |
| BOT3 | 370ϭ-I/22229 | 582.9 | 4.36 | C_48_-C_54_ | - | -27.7 | -28.3 | -0.6 | -293 | -233 | Equine adipose |
| BOT4 | 370ϭ/22250 | 61.2 | 5.35 | - | - | - | - | - | -268 | -203 | Equine adipose |
| BOT5 | 370ϭ/10886 | 67.2 | - | - | - | - | - | - | - | - | Contamination |
| BOT6 | Luϭ -84/57 | 66.6 | 1.4 | - | - | - | - | - | -228 | -199 | Animal fat |
| BOT7 | 370ϭ/43411 | 254.0 | 5.29 | - | - | -27.8 | -27.7 | 0.1 | -226 | -60 | Equine milk |
| BOT8 | 3054ϭ | 563.5 | 6.08 | C_48_-C_54_ | C_31_, C_33_ | -27.8 | -27.9 | -0.1 | -261 | -193 | Equine adipose |
| BOT9 | - | 563.8 | 3.82 | - | C_31_-C_35_ | - | - | - | - | - | Animal fat |
| BOT10 | - | 440.4 | 5.32 | - | - | -27.9 | -27.9 | 0.0 | -238 | -103 | Equine milk |
| BOT11 | 370ϭ/11721 | 63.0 | - | - | - | - | - | - | - | - | Contamination |
| BOT12 | - | 3733.1 | 0.77 | C_46_-C_56_ | - | -25.3 | -26.4 | -1.0 | -258 | -242 | Ruminant adipose |
| BOT13 | 370ϭV/6297 | 29.4 | - | - | - | - | - | - | - | - | - |
| BOT14 | - | 236.3 | 5.08 | - | C_31_-C_35_ | -27.0 | -27.6 | -0.6 | -275 | -220 | Equine adipose |
| BOT15 | 370ϭ/222320 | 54.1 | - | - | - | - | - | - | - | - | Animal fat |
| BOT16 | 255 | 333.8 | 0.82 | - | C_31_-C_35_ | -25.9 | -27.3 | -1.4 | -272 | -274 | Ruminant adipose |
| BOT17 | - | 30.2 | - | - | - | - | - | - | - | - | Contamination |
| BOT18 | 370ϭ/37959 | 510.3 | 3.72 | - | C_31_, C_33_ | -26.7 | -27.0 | -0.4 | -257 | -207 | Equine adipose |
| BOT19 | 370ϭ-I/23750 | 381.6 | 2.36 | C_48_-C_56_ | - | -26.5 | -27.2 | -0.7 | -269 | -207 | Equine adipose |
| BOT20 | 370ϭ-I/48189 | 98.6 | 4.07 | - | - | -26.4 | -26.8 | -0.3 | -268 | -214 | Equine adipose |
| BOT21 | 370ϭ/45804 | 262.7 | 2.97 | C_48_-C_54_ | - | -26.5 | -27.4 | -0.8 | -268 | -210 | Equine adipose |
| BOT22 | 370ϭ-II/19838+19814 | 202.9 | 3.15 | - | - | - | - | - | -267 | -188 | Equine adipose |
| BOT23 | 227 | 326.2 | 8.21 | - | - | -28.0 | -27.9 | 0.1 | -249 | -193 | Equine adipose |
| BOT24 | 370ϭ/6912 | 554.4 | 4.97 | - | - | -27.4 | -27.5 | -0.1 | -261 | -200 | Equine adipose |
| BOT25 | 34 | 961.2 | 0.98 | - | C_31_-C_35_ | -27.2 | -27.9 | -0.8 | -258 | -221 | Animal fat |
| BOT26 | 370ϭ-I/48356 | 605.7 | 13.25 | C_48_-C_56_ | C_31_, C_33_ | -27.3 | -27.7 | -0.4 | -250 | -179 | Equine adipose |
| BOT27 | - | 113.5 | - | - | - | - | - | - | - | - | Contamination |
| BOT28 | 370ϭ/68819 | 83.4 | - | - | - | - | - | - | - | - | Animal fat |
| BOT29 | - | 108.9 | 11.67 | - | - | - | - | - | - | - | Equine adipose |
| BOT30 | 370ϭ/3312 | 38.1 | 3.35 | - | - | - | - | - | - | - | Animal fat – likely equine |
| BOT31 | 370ϭ59933 | 17.5 | 12.54 | - | - | - | - | - | - | - | Animal fat – likely equine |
| BOT32 | - | 0.0 | - | - | - | - | - | - | - | - | - |
| BOT33 | 370ϭ/42971 | 43.9 | 3.3 | - | - | - | - | - | - | - | Animal fat – likely equine |
| BOT34 | 370ϭ-IV/5286 | 22.6 | 6.12 | - | - | - | - | - | - | - | Animal fat – likely equine |
| BOT35 | - | 242.4 | 4.69 | C_48_-C_56_ | C_31_, C_33_ | -27.1 | -27.4 | -0.3 | -268 | -190 | Equine adipose |
| BOT36 | 370ϭ-IV/16700 | 28.6 | 4.21 | - | - | - | - | - | - | - | Animal fat – likely equine |
| BOT37 | 370ϭ-IV/14418 | 56.3 | 3.56 | - | - | -29.8 | -29.0 | 0.8 | -212 | -56 | Equine milk |
| BOT38 | - | 86.3 | 3.99 | - | - | -28.6 | -27.6 | 1.0 | -234 | -170 | Equine adipose |
| BOT39 | - | 200.1 | 3.13 | - | - | - | - | - | -248 | -172 | Equine adipose |
| BOT40 | 370ϭ-IV/21579 | 55.3 | - | - | - | - | - | - | - | - | Animal fat |
| BOT41 | 370ϭ-IV/20901 | 27.0 | 2.3 | - | - | - | - | - | -229 | -176 | Equine adipose |
| BOT42 | - | 1241.0 | 0.94 | C_44_-C_56_ | - | -28.0 | -30.6 | -2.6 | -239 | -226 | Ruminant adipose |
| BOT43 | - | 40.3 | 3.49 | - | - | -28.9 | -30.3 | -1.5 | -194 | -143 | Equine adipose |
| BOT44 | /9579 | 208.5 | 4.93 | - | - | - | - | - | -274 | -191 | Equine adipose |
| BOT45 | - | 29.3 | - | - | - | - | - | - | - | - | Animal fat |
| BOT46 | 370ϭ-V/6871 | 177.1 | 6.05 | - | - | -25.7 | -26.6 | -1.0 | -248 | -193 | Equine adipose |
| BOT47 | 370ϭ-V/14777 | 1001.4 | 4.62 | C_46_-C_56_ | - | -27.9 | -29.1 | -1.2 | -272 | -186 | Equine adipose |
| BOT48 | 370ϭ-V/6041 | 191.5 | 2.63 | - | - | -28.3 | -28.5 | -0.2 | -263 | -207 | Equine adipose |
| BOT49 | 370ϭ-V/14751 | 975.6 | 2.42 | C_48_-C_56_ | - | -28.3 | -28.9 | -0.6 | -263 | -196 | Equine adipose |
| BOT50 | 370ϭ-V/14743 | 64.8 | - | - | - | - | - | - | - | - | Animal fat |
| BOT51 | 370ϭ-V/6987 | 15.9 | - | - | - | - | - | - | - | - | Animal fat |
| BOT52 | 370ϭ-V/14761 | 241.4 | 5.44 | C_48_-C_54_ | - | -28.3 | -29.3 | -1.0 | -262 | -195 | Equine adipose |
| BOT53 | - | 873.5 | 4.02 | C_48_-C_56_ | - | -27.7 | -29.1 | -1.4 | -266 | -190 | Equine adipose |
| BOT54 | 370ϭ-V/38857 | 23.7 | - | - | - | - | - | - | - | - | Animal fat |
| BOT55 | 370ϭ-V/34462 | 92.8 | 1.44 | - | - | -26.4 | -26.4 | 0.0 | -194 | -163 | Equine adipose |
| BOT56 | - | 128.8 | 2.15 | - | - | -27.0 | -28.8 | -1.8 | -249 | -192 | Animal fat |
| BOT57 | 23712 | 917.4 | 4.6 | C_48_-C_54_ | - | -27.7 | -28.7 | -1.1 | -263 | -185 | Equine adipose |
| BOT58 | - | 287.3 | 3.35 | - | - | -27.0 | -27.2 | -0.3 | -246 | -183 | Equine adipose |
| BOT59 | - | 267.4 | 4.02 | C_50_-C_54_ | - | -26.9 | -26.8 | 0.1 | -218 | -160 | Equine adipose |
| BOT60 | 370ϭ-VII/1917 | 9.4 | - | - | - | - | - | - | - | - | Animal fat |
| BOT61 | 370ϭ-VII/2115 | 76.2 | 4.62 | - | - | - | - | - | - | - | Animal fat |
| BOT62 | 370ϭ-VII/1961 | 180.6 | 4.49 | - | - | -26.3 | -27.3 | -1.0 | -246 | -185 | Equine adipose |
| BOT63 | 370ϭ-VII/1904 | 188.7 | 3.22 | - | - | -26.7 | -27.4 | -0.7 | -244 | -188 | Equine adipose |
| BOT64 | 370ϭ-VII/1869 | 0.0 | - | - | - | - | - | - | - | - | - |
| BOT65 | - | 91.1 | - | - | - | - | - | - | - | - | Animal fat |
| BOT66 | - | 1811.7 | 3.09 | C_48_-C_54_ | C_31_-C_35_ | -27.0 | -28.4 | -1.4 | -261 | -197 | Animal fat |
| BOT67 | 370ϭ-III/5485 | 0.0 | - | - | - | - | - | - | - | - | - |
| BOT68 | 370ϭ-90/3608 | 529.6 | 1.65 | - | - | -26.4 | -28.4 | -1.4 | -261 | -197 | Ruminant adipose |
| BOT69 | 370ϭ/52316 | 0.0 | - | - | - | - | - | - | - | - | - |
| BOT70 | 370ϭ/67728 | 553.8 | 3.91 | - | - | -29.3 | -29.1 | 0.2 | -227 | -83 | Equine milk |
| BOT71 | - | 785.2 | 4.32 | - | - | -27.7 | -28.8 | -1.1 | -240 | -176 | Equine adipose |
| BOT72 | 370ϭ/67150 | 406.5 | 3.5 | - | - | - | - | - | - | - | Equine adipose |
| BOT73 | - | 378.9 | 3.31 | - | - | -28.7 | -29.9 | -1.3 | -191 | -56 | Equine milk |
| BOT74 | 370ϭ-I/3877 | 450.7 | 3.64 | - | - | -28.0 | -28.9 | -1.0 | -261 | -183 | Equine adipose |
| BOT75 | - | 1128.3 | 5.49 | - | - | -27.4 | -28.3 | -0.9 | -251 | -178 | Equine adipose |
| BOT76 | 370ϭ-I/518 | 1754.1 | 1.2 | C_50_-C_54_ | - | -26.5 | -29.3 | -2.8 | -245 | -232 | Ruminant adipose |
| BOT77 | 370ϭ-I/40895 | 422.6 | 3.46 | - | C_31_, C_33_ | -28.1 | -28.5 | -0.4 | -238 | -174 | Equine adipose |
| BOT78 | - | 614.3 | 0.75 | C_46_-C_56_ | - | -27.9 | -30.5 | -2.6 | -237 | -235 | Ruminant adipose |
| BOT79 | - | 3661.2 | 1.07 | - | C_31_-C_35_ | -26.2 | -28.0 | -1.8 | -244 | -214 | Ruminant adipose |
| BOT80 | 370ϭ-I/2513Y | 0.0 | - | - | - | - | - | - | - | - | - |
| BOT81 | 370ϭ-I/30517 | 584.8 | 0.71 | C_44_-C_54_ | - | - | - | - | -221 | -235 | Ruminant adipose |
| BOT82 | - | 0.0 | - | - | - | - | - | - | - | - | - |
| BOT83 | 31590 | 15.5 | - | - | - | - | - | - | - | - | Animal fat |
| BOT84 | 370ϭ92/1771 | 102.6 | 4.33 | - | - | -27.3 | -27.9 | -0.7 | -244 | -145 | Equine adipose |
| BOT85 | 370ϭ92/1384 | 270.0 | 2.8 | C_46_-C_56_ | - | -27.4 | -28.3 | -0.9 | -254 | -172 | Equine adipose |
| BOT86 | 370ϭ92/658 | 177.0 | 3.9 | C_50_-C_54_ | - | -26.1 | -26.8 | -0.7 | -255 | -188 | Equine adipose |
| BOT87 | 370ϭ92/4244 | 82.9 | 3.49 | - | - | - | - | - | - | - | Animal fat – likely equine |
| BOT88 | - | 1127.6 | 3.56 | C_48_-C_54_ | - | -27.7 | -28.5 | -0.8 | -269 | -214 | Equine adipose |
| BOT89 | - | 384.9 | 3.82 | - | C_31_, C_33_ | -27.0 | -28.8 | -1.8 | -262 | -186 | Equine adipose |

Table S2: Summary of published radiocarbon dates from Botai.

| **Laboratory number** | **Conventional ^14^C age BP** | **Material** | **Context** | **Excavation** | **Reference** |
| --- | --- | --- | --- | --- | --- |
| OXA-4315 | 4630 ± 75 | Horse bone, 3^rd^ tarsal | Botai 26, lower part cultural horizon, 50 cm depth | 1988, Zaibert | Levine 1997 |
| OXA-4316 | 4620 ± 80 | Horse bone, distal 1rst phalange | Botai 26, Clay fill pit 5, 120 cm depth | 1988 Zaibert | Levine 1997 |
| OXA-4317 | 4630 ± 80 | Trepanned human skull, occipital bone fragment | Botai 15, caly fill pit 10, 80 cm depth | 1983, Zaibert | Levine 1997 |
| OXA-18383 | 4658 ± 33 | Horse tooth (no7) | Botai 14, 70-80cm depth | 2005 | Outram 2009 |
| UBA-32662 | 4598 ± 46 | Human crania (male) | Botai 14, burial | 1983, Zaibert | De Barros Damgaard 2018 |
| UBA-32663 | 4474 ±37 | Human crania | Botai 15, isolated find | 1983, Zaibert | De Barros Damgaard 2018 |
| UBA-32666 | 4695 ± 50 | Human (female) | Next to a house | 2016, Outram | De Barros Damgaard 2018 |
| UBA-33488 | 4693 ± 35 | Horse bone | Post hole |  | Gaunitz 2018 |
| BA121467 | 4595 ± 35 | Tree branch | B2/0095 house Zone A | 2016, Outram | Motuzaite Matuzeviciute 2019 |
| BA121469 | 4490 ± 25 | Wood charcoal | B2/0058 house Zone A | 2016, Outram | Motuzaite Matuzeviciute 2019 |
| BA121466 | 4440 ± 25 | Parenchyma | C2/0067 house Zone A | 2016, Outram | Motuzaite Matuzeviciute 2019 |
| BA120864 | 4465 ± 30 | Twig | B2/0059 house Zone A | 2016, Outram | Motuzaite Matuzeviciute 2019 |
| BA120872 | 4450 ± 30 | Tree bark | B2/0059 house Zone A | 2016, Outram | Motuzaite Matuzeviciute 2019 |
| BA120858 | 4475 ± 35 | Twig | B2/0081 house Zone A | 2016, Outram | Motuzaite Matuzeviciute 2019 |
| BA120857 | 4530 ± 35 | Twig | B2/0024 house Zone A upper floor | 2016, Outram | Motuzaite Matuzeviciute 2019 |
| BA121465 | 4385 ± 25 | Wood charcoal | B2/0035 house Zone A | 2016, Outram | Motuzaite Matuzeviciute 2019 |
| BA121471 | 4375 ± 25 | Wood charcoal | B2/0035 house Zone A | 2016, Outram | Motuzaite Matuzeviciute 2019 |
| BA121470 | 4355 ± 25 | Wood charcoal | B2/0026 house Zone A upper floor | 2016, Outram | Motuzaite Matuzeviciute 2019 |
| BA121468 | 4325 ± 25 | Wood charcoal | B2/0057 house Zone A | 2016, Outram | Motuzaite Matuzeviciute 2019 |
| IGAN-432 | 4340 ± 120 | Bone | - | Zaibert | Levine 1999 |
| IGAN-449 | 3530 ± 160 | Charcoal | - | Zaibert | Levine 1999 |
| IGAN-4234 | 4900 ± 50 | Bone | - | Zaibert | Levine 1999 |
| IGAN-4235 | 4160 ± 40 | Bone | - | Zaibert | Levine 1999 |
| IGAN-4236 | 4540 ± 60 | Bone | - | Zaibert | Levine 1999 |
| IGAN-4237 | 4430 ± 60 | Bone | - | Zaibert | Levine 1999 |

Table S3: Result table from molecular and isotopic analyses of pottery residues from Bestamak (Stear 2008).

| **Sample ID** | **Site ID** | **Concentration (µg.g^-1^)** | **C_16:0_/C_18:0_ ratio** | **TAGs** | **Ketones** | **δ^13^C_16:0_ (‰)** | **δ^13^C_18:0_ (‰)** | **Δ^13^C (‰)** | **Assignment** |
| --- | --- | --- | --- | --- | --- | --- | --- | --- | --- |
| PS452 | ПЛ/24737 | 2.5 | 3.68 | - | - | - | - | - | Animal fat - equine |
| PS453 | ПЛ/7662 | 1.4 | - | - | - | - | - | - | - |
| PS454 | ПЛ/24739 | 0 | - | - | - | - | - | - | - |
| PS455 | ПЛ/13003 | 0 | - | - | - | - | - | - | - |
| PS456 | ПЛ/1879 | 137.8 | 2 | - | - | -26.33 | -26.1 | 0.23 | Equine fat |
| PS457 | ПЛ/4084 | 0 | - | - | - | - | - | - | - |
| PS458 | ПЛ/3932 | 0 | - | - | - | - | - | - | - |
| PS459 | ПЛ/12010 | 0 | - | - | - | - | - | - | - |
| PS460 | ПЛ/10047 | 95.1 | 6.6 | - | - | -28.43 | -29.25 | -0.82 | Equine fat |
| PS461 | ПЛ/18338 | 0 | - | - | - | - | - | - | - |
| PS462 | ПЛ/11030 | 29.1 | 2.6 | - | - | -26.43 | -25.63 | 0.8 | Equine fat |
| PS463 | ПЛ/13035 | 0 | - | - | - | - | - | - | - |
| PS464 | ПЛ/21056 | 1.9 | - | - | - | - | - | - | - |
| PS465 | ПЛ/19612 | 12.4 | - | - | - | - | - | - | contamination |
| PS466 | ПЛ/15334 | 166.4 | 4.57 | - | - | -28.32 | -27.7 | 0.62 | Equine fat |
| PS467 | ПЛ/12804 | 11.7 | 2.4 | - | - | - | - | - | - |
| PS468 | ПЛ/27488 | 224.6 | 3.18 | - | - | -26.68 | -27.02 | -0.34 | - |
| PS469 | ПЛ/26895 | 144.0 | 1.75 | - | - | -27.27 | -26.87 | 0.4 | Equine fat |
| PS470 | ПЛ/1612 | 64.0 | - | - | - | - | - | - | - |
| PS471 | ПЛ/26404 | 0 | - | - | - | - | - | - | - |
| PS472 | ПЛ/7622 | 32.3 | 6.23 | - | - | -28.71 | -26.97 | 1.74 | Equine fat |
| PS473 | ПЛ/9053 | 47.3 | - | - | - | - | - | - | contamination |
| PS474 | ПЛ/7941 | 4.0 | - | - | - | - | - | - | - |
| PS475 | ПЛ/2369 | 21.6 | - | - | - | - | - | - | - |
| PS476 | ПЛ/13608 | 7.9 | - | - | - | - | - | - | - |
| PS477 | ПЛ/14162 | 33.6 | 4.15 | - | - | - | - | - | Animal fat - equine |
| PS478 | ПЛ/24287 | 4.3 | - | - | - | - | - | - | - |
| PS479 | ПЛ/17420 | 246.9 | 3.75 | - | - | - | - | - | Animal fat - equine |
| PS480 | ПЛ/20976 | 19.3 | 2.62 | - | - | - | - | - | Animal fat |
| PS481 | ПЛ/10082 | 103.2 | 1.46 | - | - | -27.33 | -27.64 | -0.31 | Equine fat |
| PS482 | ПЛ/5954 | 8.9 | - | - | - | - | - | - | Contamination |
| PS483 | ПЛ/11019 | 27.0 | - | - | - | - | - | - | Contamination |
| PS484 | ПЛ/17667 | 247.4 | 1.47 | - | - | -22.96 | -22.66 | 0.3 | Equine fat |
| PS485 | ПЛ/18952 | 0 | - | - | - | - | - | - | - |
| PS486 | ПЛ/2089 | 105.7 | 2.71 | - | - | -27.56 | -26.84 | 0.73 | Equine fat |
| PS487 | ПЛ/10825 | 55.3 | 5 | - | - | -28.93 | -27.81 | 1.12 | Equine fat |
| PS488 | ПЛ/12709 | 39.6 | 4.86 | - | - | -27.53 | -28.4 | -0.86 | Equine fat |
| PS489 | ПЛ/18139 | 53.2 | 3.79 | - | - | -28.28 | -30.32 | -2.04 | Equine fat or ruminant fat |

**
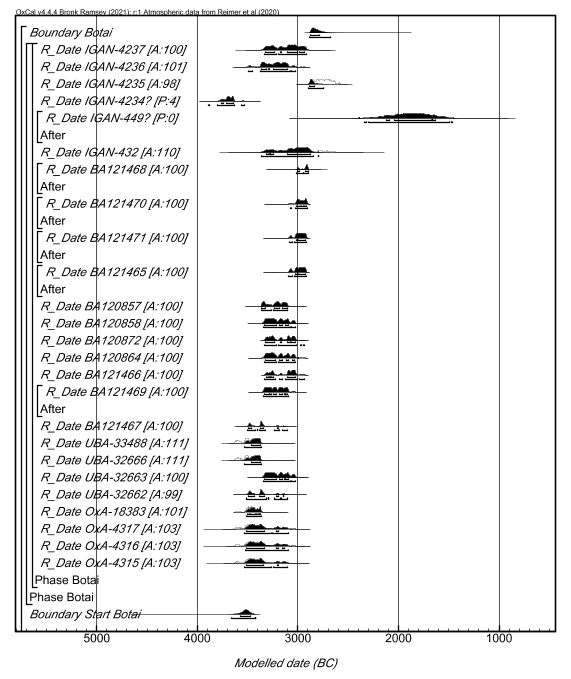
**

Figure S1: Probability distribution of published ^14^C dates for Botai. Each distribution represents the relative probability that an event occurs at a particular time. For each of the dates two distributions have been plotted: one in outline, which is the result of simple radiocarbon calibration, and a solid one, based on the chronological model used. Distributions other than those relating to particular samples correspond to aspects of the model. The distribution followed by ‘?’ has been excluded from the model. The large square brackets down the left-hand side of the figure, along with the OxCal keywords, define the overall model exactly.

**References**

de Barros Damgaard, P., Martiniano, R., Kamm, J., Moreno-Mayar, J.V., Kroonen, G., Peyrot, M., Barjamovic, G., Rasmussen, S., Zacho, C., Baimukhanov, N., Zaibert, V., Merz, V., Biddanda, A., Merz, I., Loman, V., Evdokimov, V., Usmanova, E., Hemphill, B., Seguin-Orlando, A., Yediay, F.E., Ullah, I., Sjögren, K.-G., Iversen, K.H., Choin, J., de la Fuente, C., Ilardo, M., Schroeder, H., Moiseyev, V., Gromov, A., Polyakov, A., Omura, S., Senyurt, S.Y., Ahmad, H., McKenzie, C., Margaryan, A., Hameed, A., Samad, A., Gul, N., Khokhar, M.H., Goriunova, O.I., Bazaliiskii, V.I., Novembre, J., Weber, A.W., Orlando, L., Allentoft, M.E., Nielsen, R., Kristiansen, K., Sikora, M., Outram, A.K., Durbin, R., Willerslev, E., 2018. The first horse herders and the impact of early Bronze Age steppe expansions into Asia. Science 360, eaar7711. https://doi.org/10.1126/science.aar7711

Gaunitz, C., Fages, A., Hanghøj, K., Albrechtsen, A., Khan, N., Schubert, M., Seguin-Orlando, A., Owens, I.J., Felkel, S., Bignon-Lau, O., de Barros Damgaard, P., Mittnik, A., Mohaseb, A.F., Davoudi, H., Alquraishi, S., Alfarhan, A.H., Al-Rasheid, K.A.S., Crubézy, E., Benecke, N., Olsen, S., Brown, D., Anthony, D., Massy, K., Pitulko, V., Kasparov, A., Brem, G., Hofreiter, M., Mukhtarova, G., Baimukhanov, N., Lõugas, L., Onar, V., Stockhammer, P.W., Krause, J., Boldgiv, B., Undrakhbold, S., Erdenebaatar, D., Lepetz, S., Mashkour, M., Ludwig, A., Wallner, B., Merz, V., Merz, I., Zaibert, V., Willerslev, E., Librado, P., Outram, A.K., Orlando, L., 2018. Ancient genomes revisit the ancestry of domestic and Przewalski’s horses. Science 360, 111. https://doi.org/10.1126/science.aao3297

Levine, M., Kislenko, A.M., 1997. New Eneolithic and Early Bronze Age Radiocarbon Dates for North Kazakhstan and South Siberia. Cambridge Archaeological Journal 7, 297–300. https://doi.org/10.1017/S0959774300001992

Levine, M.A., 1999. Botai and the origins of horse domestication. Journal of anthropological archaeology 18, 29–78.

Motuzaite Matuzeviciute, G., Lightfoot, E., Liu, X., Jacob, J., Outram, A.K., Zaibert, V.F., Zakharov, S., Jones, M.K., 2019. Archaeobotanical investigations at the earliest horse herder site of Botai in Kazakhstan. Archaeological and Anthropological Sciences 11, 6243–6258. https://doi.org/10.1007/s12520-019-00924-2

Outram, A.K., Stear, N.A., Bendrey, R., Olsen, S., Kasparov, A., Zaibert, V., Thorpe, N., Evershed, R.P., 2009. The Earliest Horse Harnessing and Milking. Science 323, 1332. <https://doi.org/10.1126/science.1168594>

Rieley G., 1994. Derivatization of Organic-Compounds Prior to Gas-Chromatographic Combustion-Isotope Ratio Mass-Spectrometric Analysis -Identification of Isotope Fractionation Processes. Analyst 119, 915-919.

Stear, N.A., 2008. Changing patterns of animal exploitation in the Prehistoric Eurasian steppe: an integrated molecular, stable isotopic and archaeological approach (Doctoral thesis). University of Bristol.
